# Disrupting resistance: novel therapeutic approaches to combat multidrug resistance in fusion-negative rhabdomyosarcoma

**DOI:** 10.20517/cdr.2025.145

**Published:** 2025-12-03

**Authors:** Silvia Codenotti, Francesco Marampon, Francesca Megiorni, Carlo Guglielmo Cattaneo, Stefano Gastaldello, Enrico Pozzo, Maurilio Sampaolesi, Rossella Rota, Charles Keller, Alessandro Fanzani

**Affiliations:** ^1^Department of Molecular and Translational Medicine, University of Brescia, Brescia 25123, Italy.; ^2^Department of Radiotherapy, Policlinico Umberto I, “Sapienza” University of Rome, Rome 00161, Italy.; ^3^Department of Experimental Medicine, “Sapienza” University of Rome, Rome 00161, Italy.; ^4^Department of Physiology and Pharmacology, Karolinska Institutet, Stockholm 17177, Sweden.; ^5^Department of Development and Regeneration, KU Leuven, Leuven 3000, Belgium.; ^6^Department of Hematology and Oncology, Cell and Gene Therapy, Bambino Gesù Children’s Hospital, IRCCS, Roma 00146, Italy.; ^7^Children’s Cancer Therapy Development Institute, Hillsboro, OR 97006, USA.; ^#^These authors equally contributed to this work.

**Keywords:** Rhabdomyosarcoma, RAS, PI3K, drug resistance

## Abstract

Rhabdomyosarcomas (RMS) are aggressive pediatric soft tissue tumors. The fusion-negative subtype (FN-RMS) is characterized by RAS pathway mutations and genomic instability. While standard chemotherapies - vincristine, actinomycin D, and alkylating agents - are effective against localized disease, multidrug resistance (MDR) often leads to treatment failure in relapsed and metastatic RMS. Key drivers of MDR in FN-RMS include dysregulated RAS/PI3K signaling, enhanced DNA repair, evasion of apoptosis, and alterations in drug transport and metabolism. Preclinically, vertical inhibition of the RAS/MAPK and PI3K/AKT/mTOR pathways shows promise but is limited by toxicity and compensatory feedback. Combination strategies targeting MEK, IGF1R, and PI3K, as well as epigenetic regulators and metabolic pathways, demonstrate synergistic effects. BH3 mimetics can restore apoptotic sensitivity, especially in FBW7-deficient tumors. Radiotherapy resistance is mediated through the DNA-PK–mTORC2–AKT axis, while drug transporters such as ABCB1 and SLC7A11, along with age-dependent CYP enzyme expression, affect drug bioavailability. Targeting these convergent mechanisms offers a promising therapeutic strategy to overcome resistance in FN-RMS.

## INTRODUCTION

**Figure 1:**
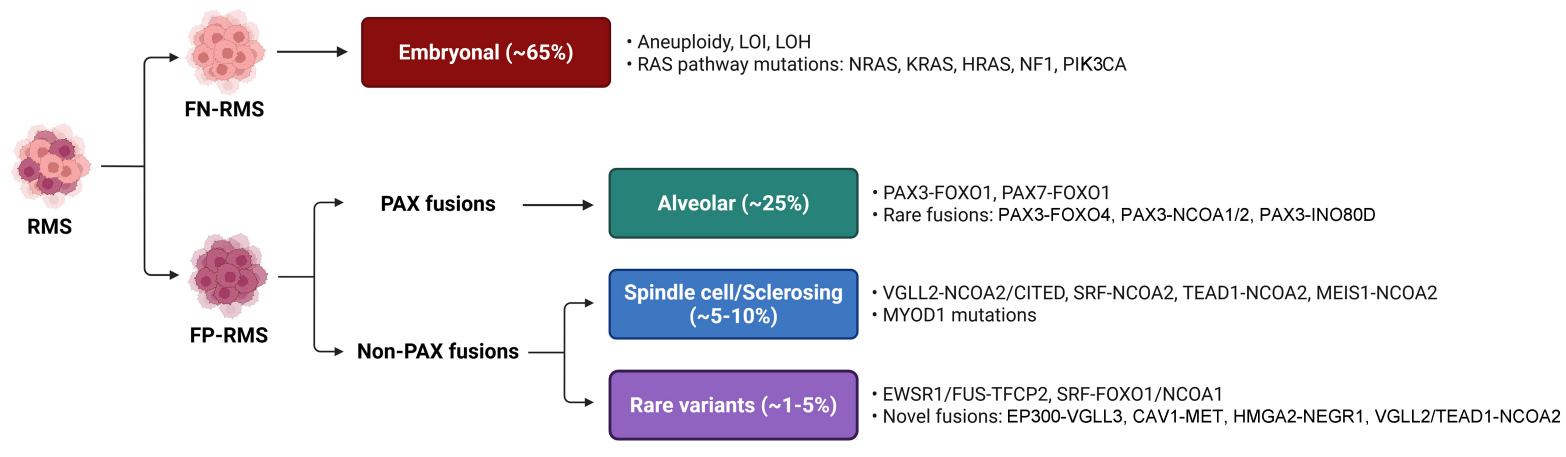
Molecular and genetic features of RMS. Primary genetic alterations and fusion oncoproteins associated with the RMS subtypes. RMS: Rhabdomyosarcomas; WHO: World Health Organization; FN-RMS: fusion-negative RMS; LOI: loss of imprinting; LOH: loss of heterozygosity; RAS: rat sarcoma; NRAS: neuroblastoma RAS viral oncogene homolog; KRAS: Kirsten rat sarcoma viral oncogene homolog; HRAS: Harvey rat sarcoma viral oncogene homolog; NF1: neurofibromin 1; PIK3CA: phosphatidylinositol-4,5-bisphosphate 3 kinase catalytic subunit alpha; FP-RMS: fusion-positive RMS; PAX: paired box gene; PAX3/7: paired box 3/7; FOXO1/4: forkhead box O1/4; NCOA1/2: nuclear receptor co-activator 1/2; INO80D: INO80 complex subunit D; VGLL2/3: vestigial-like family member 2/3; CITED: Cbp/P300 interacting transactivator with Glu/Asp rich carboxy-terminal domain 1; SRF: serum response factor; TEAD1: TEA domain family member 1; MEIS1: myeloid ecotropic viral integration site 1 homolog; MYOD1: myogenic differentiation 1; EWSR1: EWS RNA binding protein 1; FUS: fused in sarcoma; TFCP2: transcription factor CP2; CAV1: caveolin 1; MET: mesenchymal-epithelial transition factor; HMGA2: high-mobility group AT-hook 2; NEGR1: neuronal growth regulator 1.

Rhabdomyosarcomas (RMS) are high-grade malignant tumors exhibiting partial myogenic differentiation due to aberrant expression of muscle regulatory factors (MRFs) such as myogenic factor 5 (MYF5), myoblast determination protein 1 (MYOD), and myogenin (MYOG)^[1]^. Although rare in adults, RMS accounts for roughly 50% of pediatric soft tissue sarcomas, primarily affecting children, adolescents, and young adults. The World Health Organization (WHO) classifies pediatric RMS into three main histologic subtypes - embryonal, alveolar, and spindle cell/sclerosing - which display diverse morphologies and are driven by distinct genetic alterations [Figure 1].

Embryonal and alveolar RMS are the most common, representing approximately 65% and 25% of cases, respectively. Modern classification stratifies RMS into fusion-negative (FN-RMS) and fusion-positive (FP-RMS) based on the absence or presence of characteristic chromosomal translocations. FN-RMS typically exhibits complex karyotypes, aneuploidy, and somatic mutations, most commonly in rat sarcoma (RAS) pathway genes such as neuroblastoma RAS viral oncogene homolog (*NRAS*), Kirsten rat sarcoma viral oncogene homolog (*KRAS*), Harvey rat sarcoma viral oncogene homolog (*HRAS*), neurofibromin 1 (*NF1*), and phosphatidylinositol-4,5-bisphosphate 3-kinase catalytic subunit alpha (*PIK3CA*)^[2]^. Consequently, FN-RMS is considered a prototypical RAS-driven cancer^[3]^, a notion supported by the increased RMS risk in genetic syndromes such as Beckwith-Wiedemann syndrome and various RASopathies^[4]^. In contrast, FP-RMS is defined by recurrent translocations, most commonly generating paired box 3 (*PAX3*)-forkhead box O1 (*FOXO1*) or paired box 7 (*PAX7*)-*FOXO1* fusion oncoproteins, which are associated with a poorer prognosis^[2]^. It is important to note that many rare variant fusions involving *PAX3/7* or *FOXO1* with novel partner genes are being discovered in FP-RMS^[5]^. As reviews on FP-RMS and its therapeutic management are available elsewhere^[6,7]^, this review will focus primarily on novel therapeutic strategies for FN-RMS, although the rationale for some treatments may overlap across histotypes.

## MECHANISMS OF STANDARD CHEMOTHERAPY IN RMS

Multimodal treatment - combining chemotherapy, radiotherapy, and surgery - achieves durable remission in over 90% of children with low-risk, localized RMS. Standard chemotherapy regimens include VAI [vincristine, actinomycin D, ifosfamide (IFO)] or VAC [vincristine, actinomycin D, cyclophosphamide (CFO)], which demonstrate comparable efficacy in Europe and North America^[8]^. These agents act through distinct mechanisms. Vinca alkaloids (e.g., vincristine) are derived from *Catharanthus roseus* or synthesized semi-synthetically. These drugs depolymerize microtubules and destabilize the mitotic spindle, causing cell cycle arrest and apoptosis. However, resistance to vincristine often develops, making combination therapies more effective than monotherapy^[9]^.

Actinomycin D (Dactinomycin) is an antibiotic that intercalates into DNA at GpC-rich sites, inhibiting replication and blocking RNA polymerase activity, thereby impairing transcription and DNA repair. Tumor cells resistant to vincristine often show cross-resistance to actinomycin D and doxorubicin.

Alkylating agents (IFO and CFO) are prodrugs that form covalent DNA crosslinks, causing DNA damage. They require metabolic activation by cytochrome P450 (CYP) enzymes (mainly CYP3A4 and CYP2B6 for IFO)^[10]^. Consequently, co-administration of CYP3A4 inhibitors (e.g., ketoconazole, sorafenib) can reduce their efficacy.

**Figure 2:**
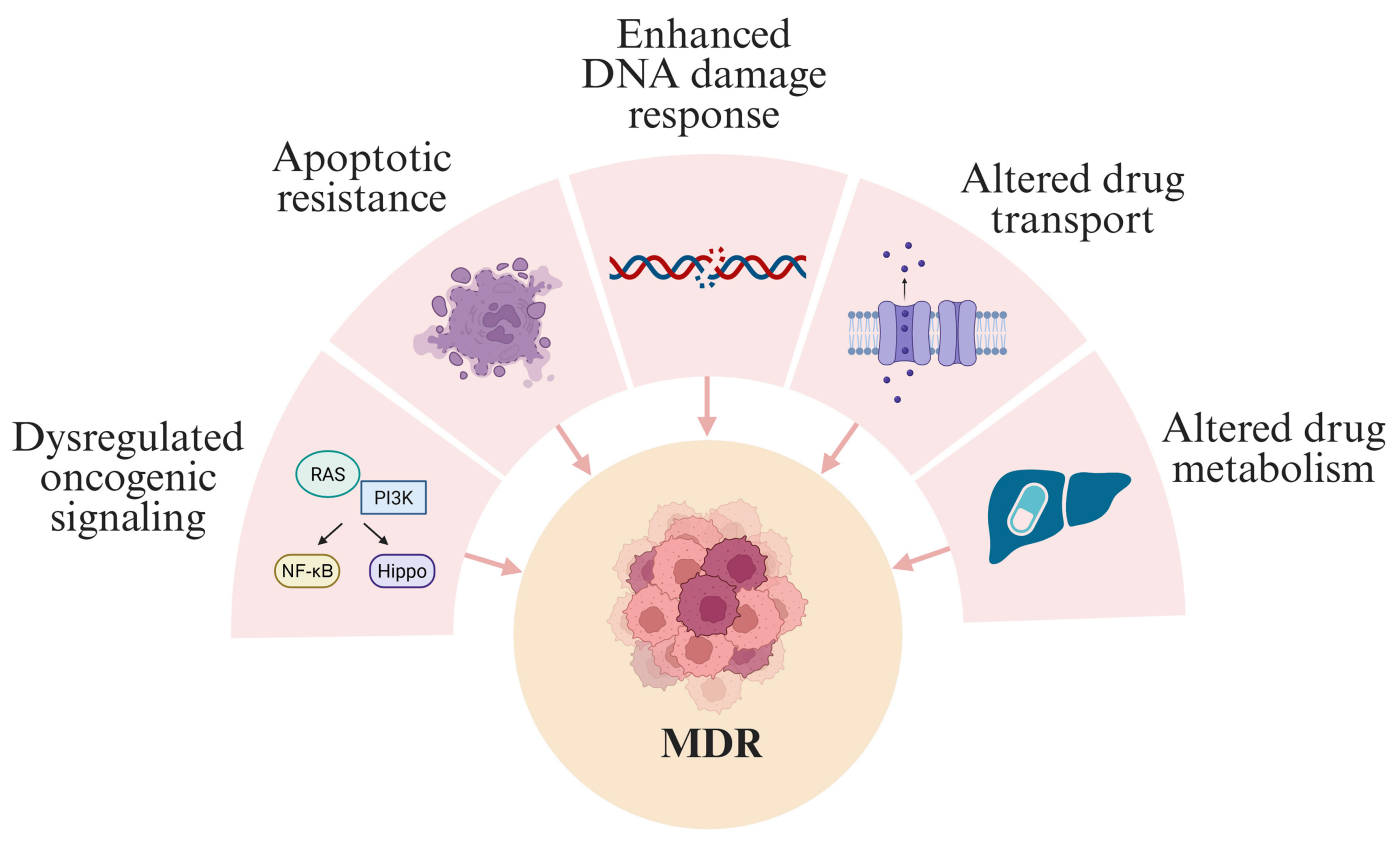
Key mechanisms leading to MDR in RMS. Several mechanisms converge in the evolution of cells with acquired MDR. MDR: Multidrug resistance; RMS: rhabdomyosarcomas; RAS: rat sarcoma; PI3K: phosphoinositide 3-kinase; NF-κB: nuclear factor kappa B.

Despite the success of these regimens in localized disease, outcomes for metastatic or relapsed RMS remain poor due to multidrug resistance (MDR)^[11-13]^. This review focuses on MDR in FN-RMS, driven primarily by dysregulated oncogenic signaling [RAS/phosphoinositide 3-kinase (PI3K)], apoptotic resistance, enhanced DNA repair, and altered drug transport and metabolism [Figure 2].

## OVERCOMING MDR IN RMS

### Vertical RAS targeting

**Figure 3:**
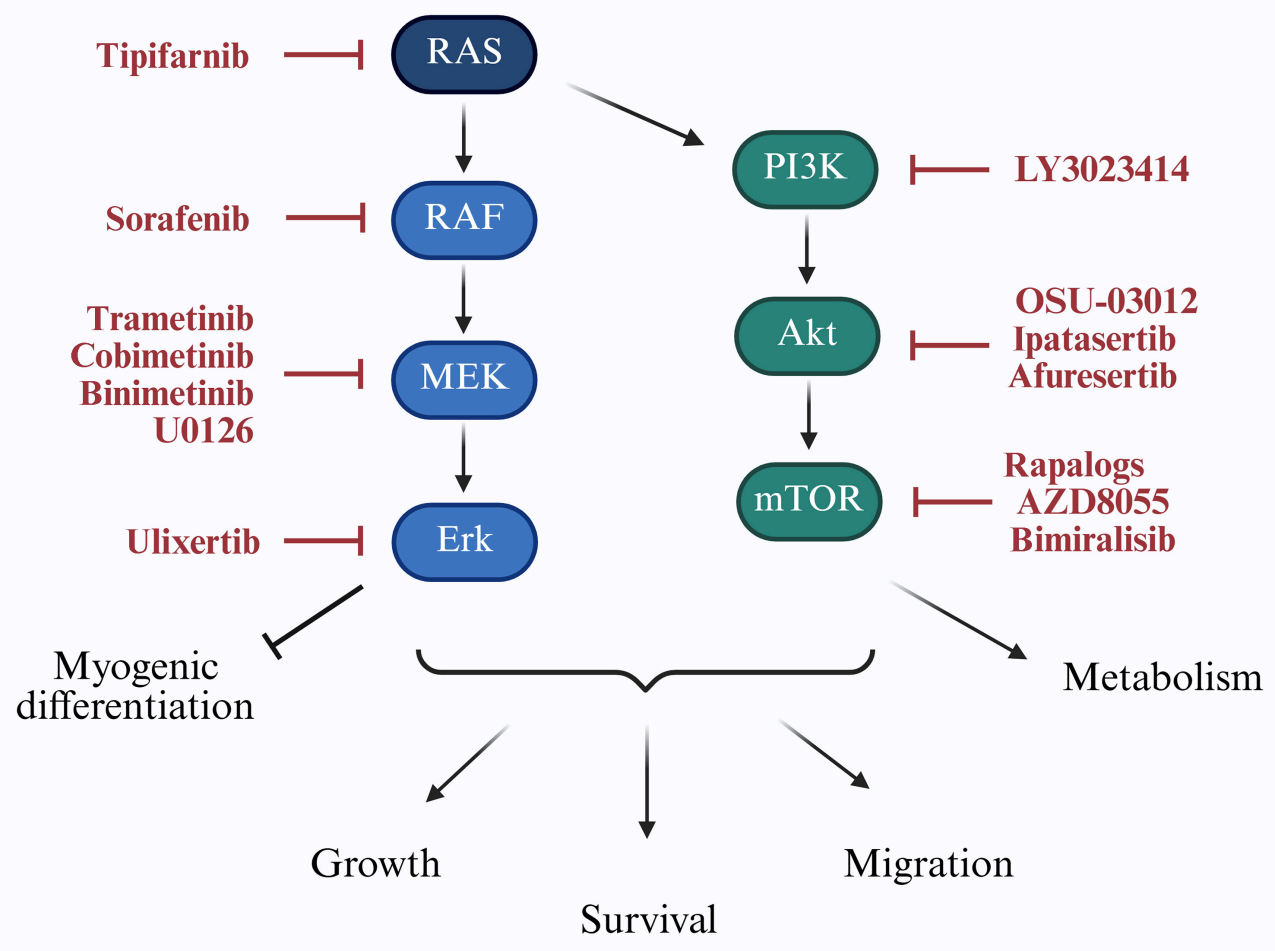
Targeting RAS/PI3K signaling in RMS. The RAS/PI3K signaling cascade regulates key cellular processes including proliferation, survival, migration, myogenic differentiation, and metabolism. Downstream effectors contribute to tumor progression and therapy resistance in RMS. Specific inhibitors targeting components of this pathway, which have been investigated in RMS models, are indicated in red. RAS: Rat sarcoma; PI3K: phosphoinositide 3-kinase; RMS: rhabdomyosarcomas; RAF: rapidly accelerated fibrosarcoma; MEK: mitogen-activated protein kinase kinase; Erk: extracellular signal-regulated kinase; Akt: protein kinase B; mTOR: mammalian target of rapamycin.

As illustrated in Figure 3, targeting the RAS/PI3K axis is a key strategy to improve RMS therapy.

The RAS family of small guanosine triphosphatases (GTPases), including HRAS, NRAS, and KRAS, regulates critical cellular functions. Oncogenic RAS mutations, which cause constitutive activation of downstream signaling, are found in approximately 25% of human cancers^[14]^. In FN-RMS, 29 distinct RAS mutations have been reported^[2]^, with isoform prevalence varying by age: HRAS in neonates, KRAS in younger children, and NRAS in adolescents^[2]^. Notably, RAS pathway activation may also be therapeutically relevant in FP-RMS^[15]^. Direct targeting of RAS has been challenging due to its compact and smooth protein structure^[16]^, leading to strategies that interfere with its membrane localization, such as with the farnesyltransferase inhibitor tipifarnib^[17]^.

Upon activation, RAS-guanosine-5′-triphosphate (GTP) stimulates the rapidly accelerated fibrosarcoma (RAF)/mitogen-activated protein kinase kinase (MEK)/extracellular signal-regulated kinase (ERK) cascade. Pan-RAF inhibitors such as sorafenib are sometimes preferred to avoid paradoxical ERK activation^[18]^. In NRAS-mutant FN-RMS models, combined RAF and MEK or ERK inhibition has induced tumor regression^[18]^ [Table 1], though sorafenib monotherapy showed limited clinical efficacy^[19]^.

**Table 1:** Combined treatments employed in preclinical RMS models

**Drug targets**	**Drugs**	**Basis for combination**	**Model**	**Endpoints**	**Risks**	**Ref.**
RAFi + MEKi	LY3009120 + trametinib	Single treatment displays little efficacy and cross-activation of compensatory pathway	RAS-mutated FN-RMS lines and tumor xenografts	Cell cycle arrest, myogenic differentiation, apoptosis, reduced tumor growth *in vivo*	Normal tissue toxicity	[18]
RAFi + Erki	LY3009120 + LY3214996					
MEKi + Erki	trametinib + LY3214996					
IGF1Ri + MEKi	BMS-754807 + trametinib	Partial response to trametinib monotherapy	RAS-mutated FN-RMS lines and tumor xenografts	Apoptosis, reduced tumor growth *in vivo*	Intolerance in murine models	[20]
IGF1Ri + MEKi	Ganitumab + trametinib	Improved tolerability compared to previous study	RAS-mutated FN-RMS lines and PDX tumor	Inhibition of cell proliferation, reduced tumor growth *in vivo*	Hematologic and metabolic side effects	[21]
MEKi + PI3Ki	MEK162 + BYL719	Single treatment displays little efficacy and cross-activation of compensatory pathway	NRAS-mutated FN-RMS lines	Apoptosis, reduced clonogenic capacity	Metabolic side effects	[22]
Erki + MCL-1i	Ulixertinib + S63845	Apoptosis restoration	RMS lines	Apoptosis, reduced clonogenic capacity	/	[26]
MEKi + PI3Ki	U0126 + PI103	Single treatment displays little efficacy and cross-activation of compensatory pathway	RMS lines	Apoptosis	/	[38]
FGFRi + HSP90i	LY2874455 + NVP-AUY922	Folding of FGFR4 V550L protein may depend on HSP90	RMS559 harboring FGFR4 V550L mutation and RH30 lines	Reduced cell viability	/	[39]
MEKi + Akti	BI-847325 + afuresertib	Single treatment displays little efficacy and cross-activation of compensatory pathway	RAS-mutant PDX-derived cells and RMS tumors	Apoptosis, reduced tumor growth *in vivo*	/	[41]
PI3Ki + BCL-2i	NVP-BKM120 + ABT-737	Apoptosis restoration	RMS lines	Apoptosis	/	[42]
mTORi + BCL-2i	AZD8055 + ABT-737					
PI3Ki + IGF1Ri	Buparlisib + NVP-AEW541	Single treatment displays little efficacy and cross-activation of compensatory pathway	RMS lines	Apoptosis	Cytotoxicity	[45]
PI3Ki + MEKi	Buparlisib + trametinib					
PI3Ki + mTORi	Buparlisib + rapamycin					
BCL-2i + NF-κBi	Navitoclax + BAY 11-7082	Apoptosis restoration	FP-RMS lines	Cytotoxic effect	/	[59]
BCL-2i + HDACi	ABT-199 + NJ-26481585	Apoptosis restoration	Primary PDX cells	Apoptosis, inhibition of long-term survival	/	[67]
MEKi + MCL-1i	trametinib + S63845	Single treatment displays little efficacy and cross-activation of compensatory pathway	PDX tumor cells	Reduced tumor growth *in vivo*	/	[68]

RMS: Rhabdomyosarcomas; RAFi: rapidly accelerated fibrosarcoma inhibitor; MEKi: mitogen-activated protein kinase kinase inhibitor; Erki: extracellular signal-regulated kinase inhibitor; RAS: rat sarcoma; FN-RMS: fusion-negative RMS; IGF1Ri: insulin-like growth factor-1 receptor inhibitor; PDX: patient-derived xenograft; PI3Ki: phosphoinositide 3-kinase inhibitor; NRAS: neuroblastoma RAS viral oncogene homolog; MCL-1i: myeloid cell leukemia-1 inhibitor; FGFRi: fibroblast growth factor receptor inhibitor; HSP90i: heat shock protein 90 inhibitor; FGFR4: fibroblast growth factor receptor 4; Akti: protein kinase B inhibitor; BCL-2i: apoptosis regulator B-cell CLL/lymphoma 2 inhibitor; mTORi: mammalian target of rapamycin inhibitor; NF-κBi: nuclear factor kappa B inhibitor; FP-RMS: fusion-positive RMS; HDACi: histone deacetylase inhibitor.

**Figure 4:**
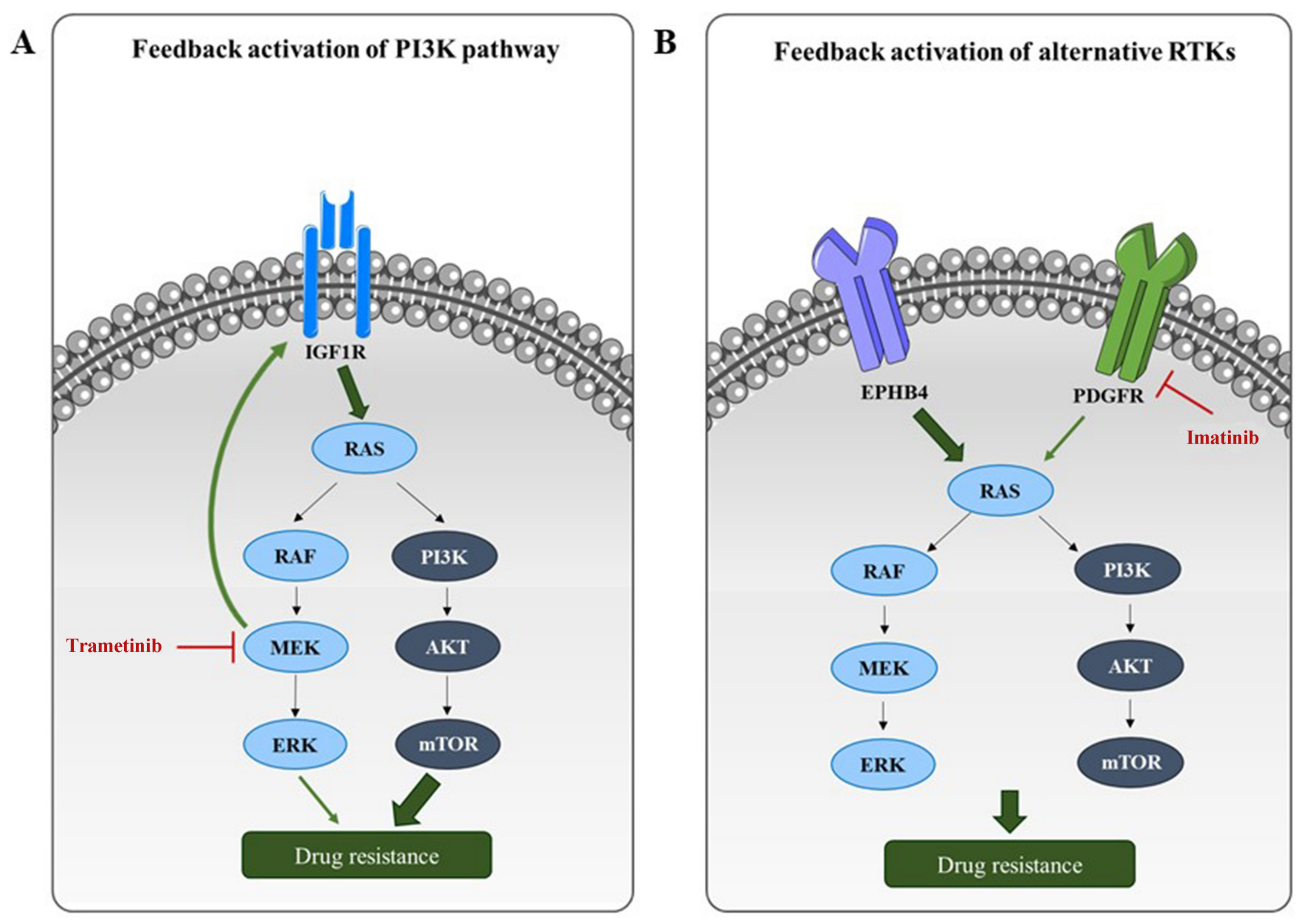
Resistance mechanisms to RAS/PI3K pathway targeting in RMS. (A) Monotherapies targeting RAS or PI3K pathway components often induce compensatory feedback mechanisms, resulting in reactivation of RAS/PI3K signaling or upstream RTKs. For example, MEK inhibition by trametinib leads to upregulation of IGF1R, which in turn reactivates the PI3K pathway; (B) Inhibition of a specific RTK can also lead to compensatory signaling through alternative RTKs. For instance, PDGFR inhibition by imatinib may be bypassed through alternative activation of the RAS/PI3K axis via EPHB4. RAS: Rat sarcoma; PI3K: phosphoinositide 3-kinase; RMS: rhabdomyosarcomas; RTKs: receptor tyrosine kinases; MEK: mitogen-activated protein kinase kinase; IGF1R: insulin-like growth factor-1 receptor; PDGFR: platelet-derived growth factor receptor; EPHB4: ephrin type-B receptor 4; RAF: rapidly accelerated fibrosarcoma; ERK: extracellular signal-regulated kinase; AKT: protein kinase B; mTOR: mammalian target of rapamycin.

MEK inhibitors (e.g., trametinib) often elicit transient responses due to compensatory activation of receptor tyrosine kinases (RTKs) and the PI3K/protein kinase B (AKT) pathway [Figure 4]. Dual inhibition of MEK and insulin-like growth factor-1 receptor (IGF1R)^[20,21]^ or PI3K^[22]^ has shown synergistic effects in xenograft models [Table 1].

Furthermore, combining trametinib with CDK4/6 inhibitors reduces viability in RAS-mutant RMS by counteracting upregulation of the cyclin D–CDK4/6–RB–E2F axis^[23]^. Resistance can also involve activation of the Hippo pathway effector yes-associated protein 1 (YAP1)^[24]^. While RAS/mitogen-activated protein kinase (MAPK) pathway inhibition has been explored with radiotherapy^[25]^, ERK inhibition alone is insufficient for apoptosis due to survival pathways such as myeloid cell leukemia-1 (MCL-1) overexpression. Combining ulixertinib (ERK inhibitor) with the MCL-1 antagonist S63845 restores apoptosis preclinically^[26]^ [Table 1], though clinical translation has so far been limited^[27]^.

Beyond proliferation and survival, the RAS/MAPK pathway suppresses myogenic differentiation^[20,28,29]^. Its pharmacological inhibition can restore differentiation and reduce tumorigenicity in preclinical models^[18,20,30]^.

### Targeting the PI3K/AKT/mTOR pathway

Aberrant activation of the PI3K/AKT/mammalian target of rapamycin (mTOR) pathway in RMS is linked to poor short-term survival and therapy resistance^[2,31]^. Among the PI3K classes, class I - comprising heterodimers of a catalytic subunit (p110α, β, γ, or δ) and a regulatory p85 subunit - is most strongly implicated in cancer. RMS frequently harbor activating mutations in *PIK3CA* (encoding p110α) and exhibit overexpression of all four class I catalytic isoforms (p110α, β, γ, and δ). Combined inhibition of p110α (alpelisib) and p110δ (idelalisib) induces apoptosis in RMS models^[32,33]^. This axis contributes to radioresistance^[34]^, hypoxia adaptation^[35]^, and DNA repair^[34]^, and enhances the transcriptional activity of PAX3-FOXO1 in FP-RMS^[36]^.

Similar to RAS pathway inhibition, PI3K monotherapy triggers compensatory MAPK activation^[37]^, necessitating dual PI3K and RAS/ERK inhibition^[38,39]^ [Table 1], though often with dose-limiting toxicity. Combining MEK inhibitors with AKT pathway inhibitors [e.g., the 3-phosphoinositide-dependent protein kinase-1 (PDK1) inhibitor OSU-03012] has shown promise in patient-derived xenograft (PDX) models^[40,41]^ [Table 1].

Newer agents such as dual PI3K/mTOR or isoform-specific PI3K inhibitors are being evaluated. The dual mTOR inhibitor AZD8055 synergized with the MEK inhibitor selumetinib^[37]^ and the B-cell lymphoma 2 homology 3 (BH3) mimetic ABT-737^[42]^ [Table 1].

mTOR functions through two distinct complexes: mTOR complex 1 (mTORC1, rapamycin-sensitive), which regulates proliferation via ribosomal p70 S6 kinase (p70S6K) activation and mTOR complex 2 (mTORC2, rapamycin-insensitive), which controls cytoskeletal dynamics and cellular invasion^[43]^. As mTORC2 predominates in primary RMS^[44]^ and is rapamycin-insensitive, it represents a critical node in disease progression.

Despite strong preclinical data, clinical trials of combinations such as PI3K/IGF1R or PI3K/MEK have shown only modest benefit^[45]^ [Table 1].

**Figure 5:**
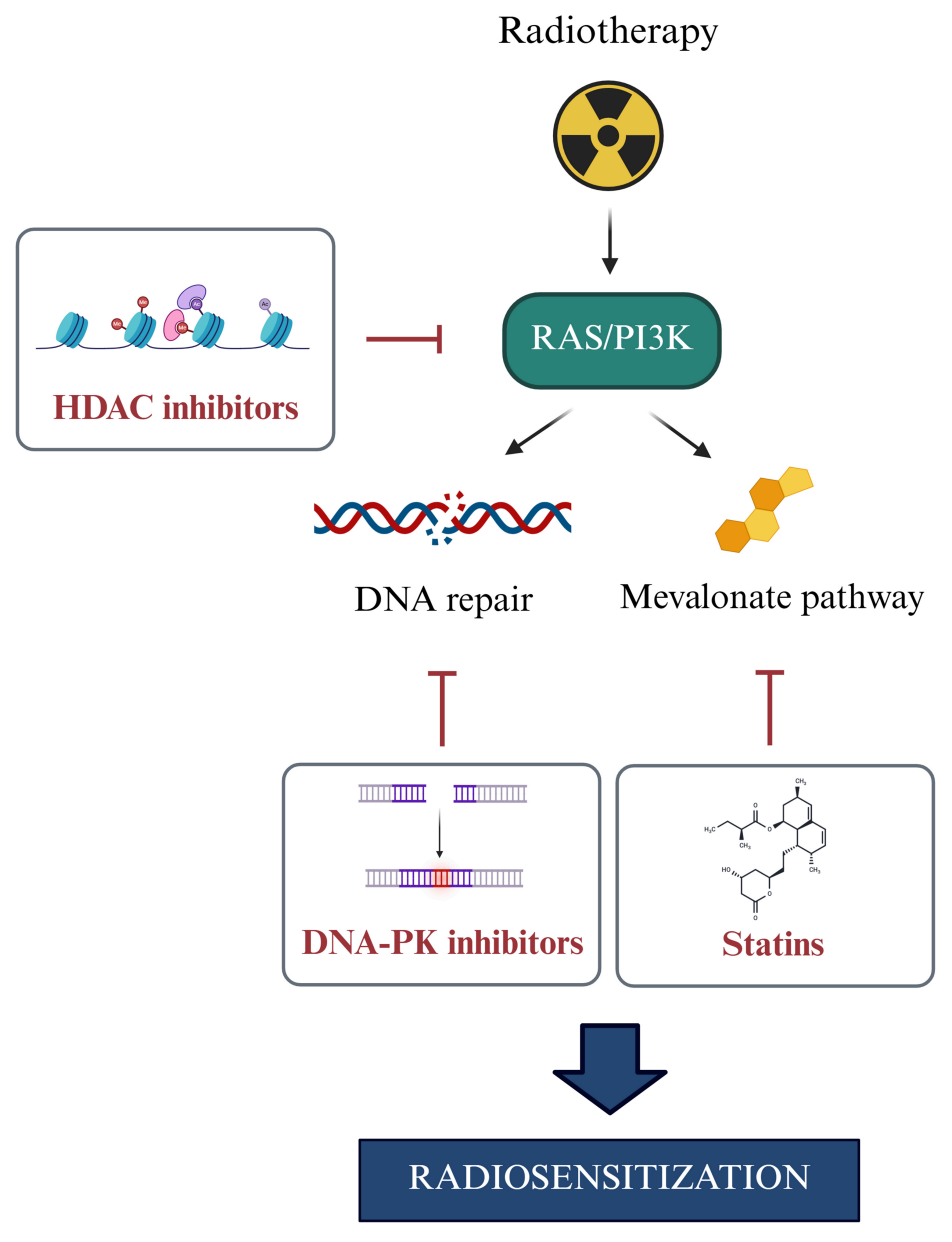
Impact of RAS/PI3K signaling on radiotherapy response. Ionizing radiation activates the RAS/PI3K signaling pathway, resulting in downstream effects that promote MDR by enhancing DNA repair and stimulating the mevalonate pathway. The use of HDAC inhibitors, DNA-PK inhibitors, or statins to target key components of this axis holds promising potential when combined with radiotherapy. RAS: Rat sarcoma; PI3K: phosphoinositide 3-kinase; MDR: multidrug resistance; HDAC: histone deacetylase; DNA-PK: DNA-dependent protein kinase.

As schematized in Figure 4, inhibition of PI3K/AKT/mTOR or RAS/MEK/ERK often triggers feedback upregulation of IGF1R [Figure 4A]^[46]^ or alternative receptors such as ephrin type-B receptor 4 (EPHB4) and platelet-derived growth factor receptor (PDGFR)^[47,48]^ [Figure 4B], supporting the need for dual targeting. New insights suggest vulnerabilities: histone deacetylase (HDAC) inhibitors^[49]^ and mevalonate pathway inhibitors^[50,51]^ can achieve radiosensitization by targeting this axis, revealing new druggable targets [Figure 5].

### Exploring alternative pathways driving MDR

Although dual PI3K and MAPK inhibition shows superior efficacy preclinically^[52]^, its clinical use is limited by toxicity^[53]^. As RMS progresses, the PI3K–AKT axis integrates with other networks, including nuclear factor kappa B (NF-κB) and Hippo pathways^[54]^.

The NF-κB pathway is a key regulator of survival and inflammation^[55]^. Oncogenic RAS activity is known to drive hyperactivation of NF-κB^[56]^, and this interaction plays a prominent role in the development of MDR. In RMS, NF-κB upregulates glycolytic enzymes such as hexokinase 2 (HK2), promoting the Warburg effect^[57]^.

Its inhibition (e.g., with curcumin) increases cytotoxicity by modulating AKT/mTOR, signal transducer and activator of transcription (STAT), 5′ adenosine monophosphate-activated protein kinase (AMPK), and p53 pathways^[58]^ and sensitizes sarcomas to navitoclax, a B cell lymphoma 2 (BCL-2) family inhibitor^[59]^ [Table 1].

The Hippo pathway, a tumor suppressor cascade, is often inactivated in RMS^[60,61]^. Its disruption leads to unchecked YAP1/TAZ (transcriptional co-activator with PDZ-binding motif) activity, promoting growth and resistance. Restoring Hippo pathway activity is therefore a viable strategy to overcome chemoresistance.

### To die or not to die: apoptotic evasion

The RAS/PI3K pathways promote therapy resistance by upregulating anti-apoptotic proteins such as BCL-2, X-linked inhibitor of apoptosis protein (XIAP), and MCL-1^[62]^, as seen in RMS^[63]^. BH3 mimetics, which inhibit these anti-apoptotic proteins, can overcome this resistance^[64]^. They show preclinical efficacy in RMS^[42,65,66]^, especially when combined with HDAC inhibitors or standard chemotherapies^[67,68]^ [Table 1].

Resistance to MEK1/2 inhibition has been linked to phorbol-12-myristate-13-acetate-induced protein 1 (PMAIP1, known as NOXA) depletion. Co-treatment with the MCL-1 inhibitor S63845 restores sensitivity to MEK inhibitors^[69]^ [Table 1].

MDR in RMS is also driven by mutations in genes such as *BCL-2*, epidermal growth factor receptor (*EGFR*), *PIK3CA*, tumor protein p53 (*TP53*), and adenosine triphosphate (ATP)-binding cassette (ABC) transporters^[70]^. A key regulatory node in this network is the tumor suppressor F-box and WD repeat domain-containing protein 7 (FBW7), a tumor suppressor that targets oncoproteins such as cellular myelocytomatosis oncogene (c-Myc) and MCL-1 for degradation. FBW7 loss stabilizes MCL-1, conferring resistance to vincristine^[71]^. As FBW7 mutations occur in ~7.4% of FN-RMS cases^[72]^, they represent a potential biomarker for predicting response to MCL-1 inhibitors or vincristine.

### DNA damage response and therapy resistance

Radiotherapy is a cornerstone of RMS treatment, but radioresistance can arise through enhanced DNA damage response (DDR). Activated AKT promotes cell survival after irradiation by facilitating repair of double-strand breaks (DSBs)^[73]^. Upon DNA damage, DNA-dependent protein kinase (DNA-PK) phosphorylates the mTORC2 subunit Sin1, leading to AKT activation and promoting survival and resistance^[74,75]^.

Both radiation and topoisomerase inhibitors can activate this DNA-PK–mTORC2–AKT axis. The PI3K/AKT and RAS/ERK pathways also contribute to DDR and resistance^[25,34]^.

Inhibiting DNA-PK catalytic subunit (DNA-PKcs) shows promise in enhancing radiosensitivity and overcoming MDR by preventing efficient DSB repair^[76]^.

### Molecular barriers to chemotherapeutic efficacy

Chemotherapy failure often results from mechanisms that limit intracellular drug accumulation or activity, involving drug transporters and metabolic enzymes. In particular, membrane transporters from the ABC, solute carrier (SLC), and solute carrier organic anion (SLCO) families play a central role in drug disposition^[77]^. While SLC and SLCO transporters mediate drug influx, ABC transporters function as efflux pumps, reducing intracellular drug concentrations and contributing significantly to MDR.

#### Efflux transporters

The human genome encodes 48 ABC transporter genes, classified into seven subfamilies (ABCA–ABCG) based on sequence homology^[78]^. Among these, ABCB1 [also known as P-glycoprotein (P-gp) or MDR1], ABCC1 [multidrug resistance protein 1 (MRP1)], and ABCG2 [breast cancer resistance protein (BCRP)] are most strongly implicated in chemoresistance^[79]^.

P-gp is a 170-kDa transmembrane efflux pump capable of exporting a wide range of chemotherapeutic agents - including vinca alkaloids, anthracyclines, and taxanes - thereby reducing their intracellular accumulation and cytotoxicity^[80]^. P-gp expression in RMS is linked to poor prognosis and actinomycin D resistance^[81,82]^. MRP1 and MDR3 (ABCB4) are upregulated post-chemotherapy, with MDR3 correlating with PAX fusions in FP-RMS^[83]^. The non-ABC transporter lung resistance-related protein (LRP) - also known as major vault protein (MVP) - also contributes to resistance by drug sequestration^[81]^.

#### Influx transporters

The SLC superfamily comprises 65 subfamilies and 458 individual transporters, which mediate the cellular uptake of ions, nutrients, and xenobiotics, including several chemotherapeutic agents^[84]^. The SLCO superfamily, consisting of six subfamilies, is responsible for the uptake of larger molecules (> 300 Da) such as bile acids, hormones, and certain chemotherapeutic drugs^[85]^. However, their role in RMS is poorly characterized, except for SLC family 7 member 11 (SLC7A11) (xCT), a cystine/glutamate antiporter that maintains glutathione synthesis and redox homeostasis, supporting resistance to oxidative stress. Often overexpressed in tumors, SLC7A11 contributes to ferroptosis resistance^[86]^. In RMS models, pharmacological inhibition of SLC7A11 triggers ferroptotic death^[87]^.

#### Drug metabolism and detoxification

Many drugs are metabolically activated or inactivated by CYP enzymes. The expression of CYP enzymes varies with age, tissue type, and developmental stage, making drug metabolism especially complex in pediatric cancers such as RMS^[88]^.

The human genome encodes 57 CYP genes across 18 subfamilies that metabolize a wide array of xenobiotics^[89]^. CYP3A4 and CYP3A5, for instance, detoxify vincristine^[90,91]^. The activity of these enzymes is age-dependent, affecting efficacy in pediatric patients.

Co-administration of CYP3A4 inhibitors can impair the activation of prodrugs such as IFO and CFO.

Notably, RMS tumors often overexpress the fetal-type CYP2W1, a fetal-type monooxygenase typically silenced after birth^[92,93]^, a potential therapeutic vulnerability.

Therapeutic strategies co-targeting CYP enzymes, transporters, and oncogenic pathways may help overcome these resistance mechanisms.

## CONCLUSIONS

**Figure 6:**
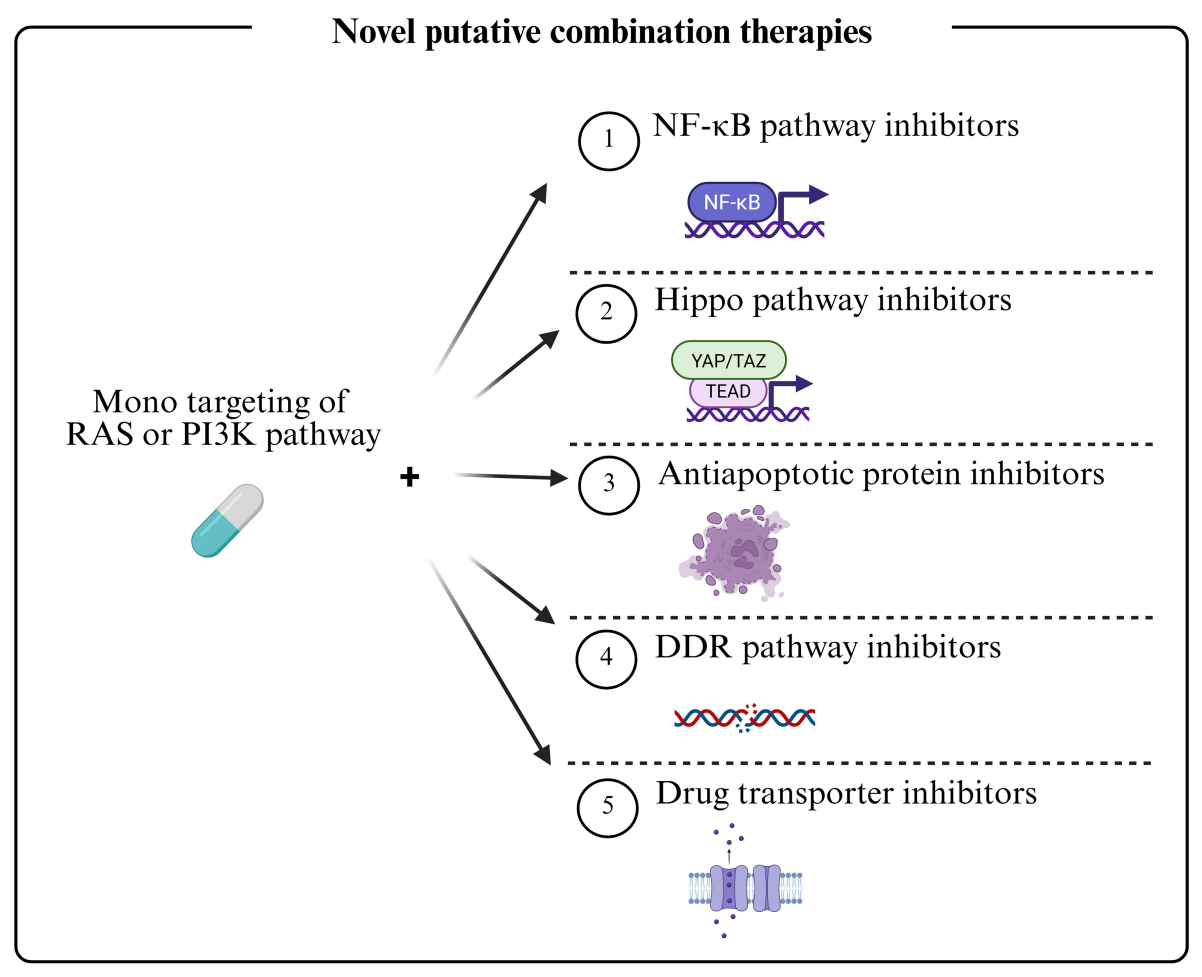
Combined therapeutic regimens against RMS. Dual inhibition of the RAS and PI3K pathways has generally proven ineffective due to the emergence of MDR mechanisms and is further limited in clinical settings by toxicity. More promising therapeutic approaches involve monotargeting of either the RAS or PI3K pathway in combination with agents targeting downstream effectors - such as those involved in alternative oncogenic signaling, apoptotic resistance, DDR and drug transport. These combined strategies, aimed at overcoming MDR in RMS, include both ongoing therapies and emerging experimental approaches. RMS: Rhabdomyosarcomas; RAS: rat sarcoma; PI3K: phosphoinositide 3-kinase; MDR: multidrug resistance; DDR: DNA damage response; NF-κB: nuclear factor kappa B; YAP: yes-associated protein; TAZ: transcriptional co-activator with PDZ-binding motif; TEAD: TEA domain family member.

Dysregulation of the RAS and PI3K pathways is central to therapy resistance in FN-RMS. While current chemotherapy is effective for localized disease, MDR causes failure in metastatic and relapsed cases. Vertical inhibition of these pathways can restore sensitivity but is limited by toxicity and compensatory feedback loops. Combination strategies targeting multiple resistance mechanisms, including signaling feedback, apoptotic evasion, DDR, and drug transport, are therefore essential [Figure 6]. For instance, BH3 mimetics or FBW7-targeting approaches can reverse apoptotic resistance. Inhibiting DNA-PK or mTORC2 can enhance radiosensitivity. Epigenetic and metabolic modulators (e.g., HDAC inhibitors, statins) show efficacy as radiosensitizers. Furthermore, efflux pumps (ABCB1, MRP1) and influx transporters (SLC7A11) significantly impact drug bioavailability, while age-dependent CYP metabolism and tumor-specific isoforms such as CYP2W1 represent metabolic vulnerabilities. To advance the field, future efforts should focus on identifying novel biomarkers^[94,95]^, developing robust preclinical models^[96]^, and leveraging machine learning for integrative analysis and predictive modeling^[97]^. A multi-faceted approach is crucial to overcome MDR and improve outcomes for patients with high-risk RMS.

## DECLARATIONS

### Copyright

© The Author(s) 2025.
